# Advancements and Challenges in Salivary Metabolomics for Early Detection and Monitoring of Systemic Diseases

**DOI:** 10.1002/mco2.70395

**Published:** 2025-09-21

**Authors:** Xinyuan Zhao, Xu Chen, Zihao Zhou, Jiarong Zheng, Yunfan Lin, Yucheng Zheng, Rongwei Xu, Shen Hu, Li Cui

**Affiliations:** ^1^ School of Stomatology Stomatological Hospital Southern Medical University Guangzhou Guangdong China; ^2^ Department of Dentistry The First Affiliated Hospital, Sun Yat‐Sen University Guangzhou China; ^3^ School of Dentistry Jonsson Comprehensive Cancer Center California NanoSystems Institute University of California Los Angeles California USA

**Keywords:** biomarkers, metabolic profiling, noninvasive diagnosis, salivary metabolomics, systemic diseases

## Abstract

Salivary metabolomics is increasingly recognized as a powerful, noninvasive approach for studying human health and disease. Unlike blood or urine, saliva is easily accessible, minimally invasive, and suitable for repeated sampling. Advances in nuclear magnetic resonance, mass spectrometry, capillary electrophoresis, and bioinformatics have improved the sensitivity and reproducibility of salivary metabolite profiling, enabling its use across diverse systemic diseases such as cancer, cardiovascular disorders, diabetes, viral infections, autoimmune diseases, and neurodegenerative conditions. Despite this progress, clinical translation is limited by variability in sampling, lack of standardized protocols, and insufficient large‐scale validation. This review synthesizes recent developments in human salivary metabolomics, emphasizing disease‐specific biomarkers and key applications in systemic disease diagnosis and monitoring. We also examine methodological and biological factors that influence data reliability, including collection methods, storage conditions, circadian rhythms, age, and host–microbiome interactions. Furthermore, integration of multiomics strategies, machine learning, and clinical registry data is discussed as a means to enhance biomarker discovery and translational potential. By addressing these challenges, salivary metabolomics can evolve into a reliable platform for noninvasive diagnosis, longitudinal disease monitoring, and personalized medicine, providing a valuable complement to blood‐based diagnostics in precision healthcare.

## Graphical abstract

Salivary metabolomics offers a noninvasive and effective approach for early disease detection and monitoring. Salivary metabolites reflect systemic metabolism, inflammation, and external factors such as diet and stress. Advance in bioinformatics and machine learning facilitates the identification of metabolic biomarkers with high diagnostic accuracy. This approach enables continuous health monitoring and differentiation of disease states, demonstrating broad potential for various systemic conditions.

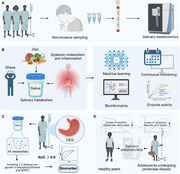

## 1 Introduction

Metabolomics, first introduced in the late 1990s, has matured into a core pillar of systems biology by enabling the comprehensive, quantitative profiling of small‐molecule metabolites in biological specimens [1, 2, 3]. Originally applied to conventional biofluids such as plasma and urine, the field has rapidly expanded to encompass tissues, cells, exhaled breath, and saliva, thereby capturing biochemical readouts closely aligned with phenotype [4, 5, 6]. By integrating the downstream consequences of genomic, transcriptomic, proteomic, microbial, and environmental influences, metabolomics provides a dynamic window into physiological states, disease pathogenesis, and therapeutic responses. Over the past two decades, the focus has shifted from proof‐of‐concept biomarker discovery to translational objectives, including early diagnosis, risk stratification, therapeutic monitoring, and individualized treatment optimization [7, 8].

**FIGURE 1:**
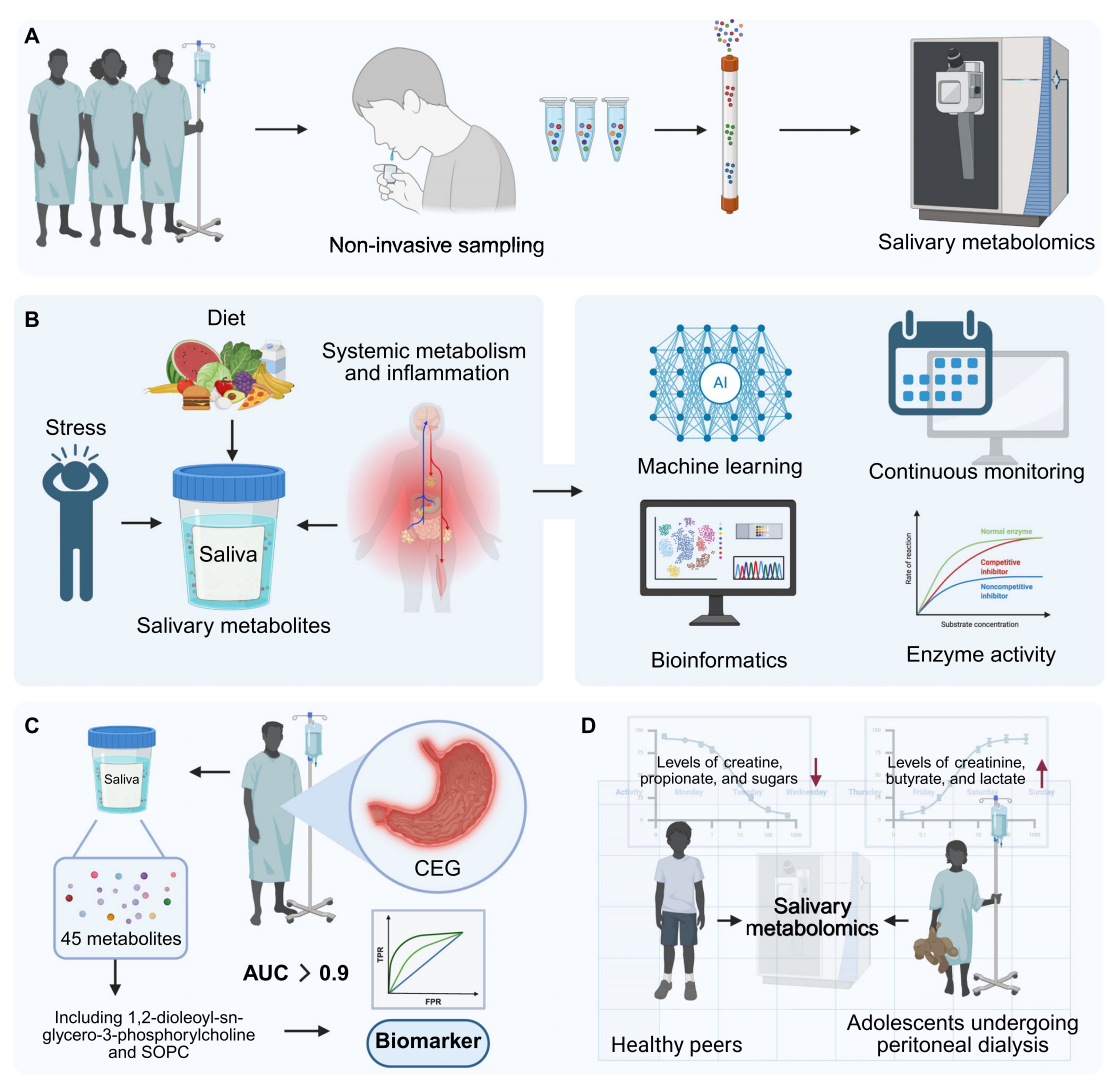
Salivary metabolomics as a noninvasive diagnostic tool for systemic diseases. (A) Illustration of saliva collection highlights its advantages as a noninvasive and convenient method for early disease detection, suitable for both clinical and home use. (B) Interactions between systemic metabolism, inflammation, and lifestyle factors such as diet and stress are shown to shape salivary metabolite profiles, with bioinformatics and machine learning approaches facilitating biomarker discovery and continuous monitoring. (C) Identification of 45 metabolites in CEG, including 1,2‐dioleoyl‐sn‐glycero‐3‐phosphocholine and SOPC, demonstrates the diagnostic accuracy of salivary metabolomics. (D) Comparative analyses across populations, such as adolescents on peritoneal dialysis versus healthy peers, underscore its potential to evaluate enzyme activity and treatment responses in diverse patient groups. Created in https://BioRender.com.

Within this expanding landscape, saliva has emerged as a particularly promising diagnostic medium. Historically underutilized due to concerns over variability, enzymatic activity, and relatively low metabolite abundance, saliva is now recognized for its noninvasive, painless, and repeatable collection; minimal reliance on trained personnel; and suitability for population‐level screening or home‐based sampling [9, 10, 11] (Figure 1A). Crucially, salivary profiles reflect both local oral conditions and systemic physiology, conferring a dual‐informative readout for health surveillance [12, 13, 14]. Advances in high‐resolution analytical platforms—nuclear magnetic resonance (NMR), gas/liquid chromatography (LC)–mass spectrometry (MS), and capillary electrophoresis (CE)—together with robust bioinformatics and machine‐learning pipelines, have substantially improved the sensitivity, depth, and reproducibility of salivary metabolomic profiling, enabling applications across metabolic disorders, infections, autoimmune conditions, malignancies, and neurodegenerative diseases [15, 16, 17] (Figure 1B).

Despite rapid methodological and conceptual progress, important barriers continue to limit clinical adoption. Chief among these are preanalytical and analytical variability (sampling method and timing, storage and preservation, batch effects), biological heterogeneity (age, sex, circadian rhythm, diet, oral hygiene, medication), and incomplete understanding of host–microbiome interactions that shape salivary chemistry [18, 19]. The lack of harmonized operating procedures and large, multicenter validation cohorts further constrains cross‐study comparability and the establishment of clinically qualified biomarkers. Addressing these gaps is essential to move salivary metabolomics from promising research toward routine clinical decision support.

Accordingly, this review provides an up‐to‐date and critical synthesis of human‐focused salivary metabolomics. We summarize disease‐specific applications spanning cancer, cardiovascular disease, diabetes, viral infections, autoimmune disorders (e.g., Sjögren's syndrome [SS]), and neurodegeneration (e.g., Alzheimer's and Parkinson's disease [AD and PD]), emphasizing diagnostic and prognostic biomarkers and their mechanistic underpinnings. We examine determinants of data quality and precision—including sampling strategies (unstimulated vs. stimulated saliva; spitting vs. aspiration), preservation and storage (e.g., inhibitors, freezing), analytical platforms (NMR, LC/GC–MS, CE–MS), and confounders such as circadian and behavioral factors. We further highlight integrative strategies that enhance translational value, including multiomics convergence (metagenomics, proteomics, lipidomics), advanced machine‐learning for feature selection and risk modeling, and incorporation of clinical registry or real‐world data to support external validation and clinical utility assessment.

The article is organized as follows. First, we outline the distinctive advantages and current technological capabilities of salivary metabolomics. Second, we review applications across major systemic disease areas, focusing on clinically relevant biomarkers and use cases in diagnosis, monitoring, and therapeutic evaluation. Third, we analyze preanalytical, analytical, and biological factors that influence data reliability and interpretability. Fourth, we discuss translational barriers and propose practical solutions—standardized protocols, quality‐control frameworks, multicenter validation, and regulatory‐aligned study designs. Finally, we present future directions for integrating salivary metabolomics into precision medicine, including multiomics fusion, deployable analytical workflows, and pathways toward clinical qualification and adoption.

## 2 Literature Search Methodology

To support the preparation of this narrative review, a comprehensive literature search was conducted using the PubMed database. The search covered publications from January 2010 to March 2025 and utilized combinations of the following keywords: “*salivary metabolomics*,” “*systemic diseases*,” “*biomarkers*,” “*noninvasive diagnosis*,” “*cancer*,” “*diabetes*,” “*neurodegenerative disorders*,” “*autoimmune diseases*,” and “*viral infections*.” Only English‐language, peer‐reviewed articles were considered. Studies were included if they focused on the application of salivary metabolomics in the diagnosis, monitoring, or pathophysiological understanding of systemic diseases. Reviews, editorials, conference abstracts, and studies unrelated to salivary analysis or systemic conditions were excluded. Reference lists of selected articles were also screened manually to identify additional relevant publications. This approach was designed to ensure a representative and up‐to‐date synthesis of the current research while minimizing selection bias.

## 3 Comparison of Blood and Saliva in Metabolomics for Systemic Diseases

Among various biological fluids, blood—particularly plasma and serum—remains the most extensively characterized in metabolomics research due to its systemic representativeness, metabolic diversity, and relative biochemical stability  [20, 21]. The blood metabolome encompasses a wide range of metabolites derived from multiple tissues and organs, including amino acids, lipids, sugars, organic acids, and hormones, many of which are well established as disease biomarkers [22, 23, 24]. Moreover, plasma metabolites exhibit high biochemical stability, tolerating repeated freeze–thaw cycles and long‐term storage with minimal degradation [25].

In contrast, the salivary exometabolome is compositionally and functionally distinct. Saliva typically contains 300–500 identifiable metabolites—fewer than plasma—but shows greater enrichment in microbial‐derived compounds, short‐chain fatty acids, nucleosides, and volatile organic compounds (VOCs) [26]. Additionally, the salivary exometabolome reflects a complex interplay between systemic circulation, local glandular secretion, and the oral microenvironment [27]. Metabolites present in saliva originate from multiple sources, including passive diffusion or active transport from blood across the salivary gland epithelium, direct synthesis and secretion by acinar and ductal cells, and microbial metabolism within the oral cavity [28]. This dual origin enables saliva to capture not only systemic biochemical alterations but also dynamic, site‐specific changes in mucosal immunity and microbiome–host interactions, which are often attenuated in blood. For example, chronic erosive gastritis (CEG) is linked to gastric cancer, necessitating noninvasive diagnostic methods. Differential metabolite analysis of saliva from CEG patients identified 45 metabolites, including 1,2‐dioleoyl‐sn‐glycero‐3‐phosphorylcholine and 1‐stearoyl‐2‐oleoyl‐sn‐glycoro‐3‐phospholine (SOPC), which showed high potential as biomarkers with area under curve (AUC) values above 0.9. These metabolites are associated with amino acid, lipid, phenylalanine metabolism, and the mTOR signaling pathway, suggesting their clinical applicability for early CEG detection [29] (Figure 1C). Similarly, salivary metabolomics reveals distinct profiles in children and adolescents undergoing peritoneal dialysis, with reduced levels of creatine, propionate, and sugars and increased levels of creatinine, butyrate, and lactate compared with healthy peers. These metabolic changes highlight the potential of salivary biomarkers for monitoring complications related to pediatric nephrology and advancing early diagnosis and management strategies in pediatric nephrology [30] (Figure 1D). However, the salivary metabolome is more vulnerable to enzymatic degradation (e.g., by amylase, proteases) and microbial activity, necessitating rigorous preanalytical standardization to ensure data reliability [31].

Despite these differences, saliva shares several technical and biological features with blood. Both biofluids contain metabolites involved in core metabolic pathways such as amino acid, energy, and lipid metabolism [32, 33]. Cross‐matrix studies have revealed considerable overlap; for example, ^1^H‐NMR analyses have identified over 30 metabolites consistently present in both matrices, underscoring their complementary diagnostic potential [34]. Moreover, both fluids are compatible with mainstream metabolomic platforms and benefit from advanced computational tools for biomarker discovery and metabolic pathway analysis [21].

Beyond its biochemical composition, saliva offers distinct practical advantages, including noninvasive, repeatable collection, minimal need for trained personnel, and suitability for point‐of‐care or home‐based testing. Unlike blood or other biofluids, saliva can be collected without the need for needles or invasive procedures, reducing patient discomfort and minimizing risks associated with sample collection [35]. This ease of collection makes it ideal for frequent sampling, which is essential in dynamic disease monitoring or longitudinal studies. Furthermore, saliva collection requires little to no specialized equipment or professional expertise, making it a cost‐effective and accessible option for both clinical settings and remote monitoring. These attributes also enhance patient compliance, particularly in populations such as children, the elderly, or individuals in rural or underserved areas [36]. As such, salivary metabolomics should be viewed as a valuable complement to blood‐based approaches, particularly advantageous for dynamic monitoring and settings requiring high accessibility and patient compliance in systemic disease contexts.

## 4 Analytical Techniques and Computational Tools in Salivary Metabolomics

Metabolomics employs a variety of analytical platforms to probe the complex biochemical signatures within biological samples, each offering distinct advantages and presenting unique challenges. For salivary metabolomics, the key technologies in use are NMR spectroscopy, LC–MS, and GC–MS. Each of these techniques has demonstrated significant potential for providing insights into the salivary metabolome, but they also come with inherent limitations that must be carefully considered when applied to complex biofluids like saliva.

NMR spectroscopy is an ideal technique for nondestructive, high‐throughput, and reproducible analysis of metabolites in saliva. NMR provides detailed chemical structural information and quantitative data on metabolites without the need for extensive sample preparation [37, 38]. This is particularly valuable in clinical settings where sample preservation and minimal disruption are essential. However, NMR's sensitivity, while suitable for detecting abundant metabolites, can be a limitation when it comes to detecting metabolites present in lower concentrations, which are often crucial for identifying disease‐specific biomarkers [39, 40]. NMR also struggles with the analysis of complex mixtures, such as those in saliva, where overlapping resonances and the presence of many metabolites can make identification difficult [41]. Despite these challenges, NMR is invaluable for initial screenings of broad metabolic classes and for monitoring changes in known metabolite classes, such as amino acids, lipids, and sugars. Moreover, recent advances in multivariate statistical analysis and machine learning algorithms have helped overcome some of NMR's limitations, allowing for more refined interpretations of complex data sets and the identification of subtle disease‐related metabolic shifts [42, 43, 44].

LC–MS is regarded as one of the gold standards in modern metabolomics due to its ability to detect a wide variety of metabolites, including lipids, amino acids, organic acids, and nucleotides, with high sensitivity and specificity [45, 46, 47]. LC–MS integrates the separation capability of LC with the MS's powerful analytical capabilities, allowing for precise identification and quantification of metabolites over a wide dynamic range [48]. This method excels in detecting low‐concentration metabolites and has become indispensable for profiling complex biological samples like saliva [49]. However, the use of LC–MS in salivary metabolomics is complicated by the presence of matrix effects, where certain components of saliva can interfere with ionization and detection, affecting the accuracy of metabolite quantification [50]. Additionally, sample preparation for LC–MS can be resource‐intensive and prone to variability, especially when salivary samples contain diverse metabolites from both the systemic circulation and local sources, such as the oral microbiota. Further optimization of protocols and advances in ionization techniques, such as electrospray ionization, have mitigated some of these issues, but they still require careful attention when comparing results across studies. LC–MS is particularly valuable for obtaining a broad spectrum of data from saliva and for linking systemic diseases with specific metabolic signatures.

GC–MS is another widely used technique for analyzing volatile metabolites and other small molecules that are typically found in biofluids, such as short‐chain fatty acids, alcohols, and aldehydes [51, 52, 53, 54]. GC–MS offers high resolution and sensitivity, making it ideal for profiling VOCs in saliva. This technique excels in applications where small, volatile metabolites are of interest and is often used in environmental and clinical studies to detect exhaled metabolites [55, 56, 57]. However, the use of GC–MS in salivary metabolomics is somewhat limited due to its reliance on sample derivatization, which introduces additional complexity and variability to the analysis. Derivatization is necessary to enhance the volatility of certain metabolites and improve their detectability by the GC–MS system [58, 59, 60]. This process can lead to changes in metabolite concentration or introduce artifacts that may affect reproducibility. Despite these challenges, GC–MS remains a valuable tool in specific contexts where detailed profiling of VOCs or hydrophobic metabolites is needed, such as in the study of metabolic disorders, cancer, or respiratory diseases [61, 62]. Beyond these platforms, recent advances in metabolomic analysis have been driven by the integration of cutting‐edge technologies such as high‐resolution MS, CE, and ambient ionization techniques. These innovations allow for more precise and sensitive measurements of metabolites in biofluids like saliva, enhancing their diagnostic utility in clinical applications.

Emerging technologies and the integration of machine learning and bioinformatics are increasingly playing a crucial role in overcoming the limitations of traditional analytical techniques in salivary metabolomics [63, 64, 65, 66]. These advanced tools enable the analysis of complex, large‐scale metabolomic datasets, uncovering subtle yet significant metabolic signatures that are linked to specific diseases [67, 68, 69]. For example, in primary SS, a combined approach using metabolomics and machine learning identified a distinct metabolic signature of six salivary metabolites, including kynurenine and phospholipids, with high discriminatory power. This signature effectively differentiated early‐stage SS from other causes of sicca symptoms and provided insights into the underlying immune‐metabolic pathways [70]. Similarly, in colorectal cancer (CRC) detection, an alternative decision tree (C)‐based machine learning method applied to saliva metabolites demonstrated strong accuracy in distinguishing CRC from adenoma and healthy controls, further underscoring the diagnostic potential of salivary metabolomics when coupled with computational tools [71].

## 5 Salivary Metabolomics for Early Detection and Monitoring of Systemic Diseases

Although saliva is produced locally, its metabolomic profile is shaped by a dynamic interplay between systemic and local physiological processes [72, 73, 74]. Systemic metabolites reach saliva through multiple well‐characterized mechanisms, including passive diffusion, active transport, and ultrafiltration from the bloodstream across the salivary gland epithelium [75]. This enables a wide range of blood‐borne small molecules—such as amino acids, urea, steroid hormones, xenobiotics, and certain lipid derivatives—to be incorporated into the salivary milieu [76]. More importantly, systemic pathological states often trigger immunological, endocrine, and metabolic cascades that exert downstream effects on the oral microenvironment [77]. For instance, chronic systemic inflammation can alter salivary gland function, modulate the composition of the oral microbiome, and disrupt mucosal barrier integrity, all of which secondarily reshape the salivary metabolome [78, 79]. Additionally, conditions such as metabolic syndrome, cancer, and neurodegenerative disorders have been shown to perturb circulating metabolite levels and inflammatory mediators that ultimately manifest in altered salivary profiles [80]. These changes are not random but occur in disease‐specific patterns that can be captured through high‐resolution metabolomic analysis. Therefore, rather than serving solely as a reflection of local oral conditions, this systemic–salivary linkage supports the clinical utility of salivary metabolomics for early detection and monitoring of systemic diseases.

### 5.1 Viral Diseases

Saliva metabolomics is particularly effective for detecting biomarkers in viral infections, including COVID‐19, due to the direct involvement of the oral mucosa in the viral infection process [81, 82, 83, 84]. Viruses like SARS‐CoV‐2 and HIV induce localized inflammation and systemic metabolic disruptions, reflected in salivary metabolite changes [85, 86, 87, 88]. Additionally, viral infections can alter the oral microbiome, contributing to specific metabolites in saliva. Given saliva's direct connection to both local and systemic changes, it serves as an ideal noninvasive medium for monitoring viral infections, tracking disease progression, and assessing treatment efficacy.

#### 5.1.1 COVID‐19

**FIGURE 2:**
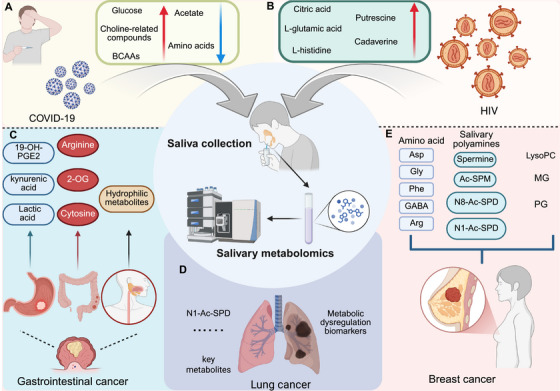
Applications of salivary metabolomics in viral infections and cancers. (A) In COVID‐19, elevated salivary glucose, choline‐related compounds, and BCAAs are accompanied by reduced acetate and amino acids, reflecting systemic metabolic reprogramming. (B) In HIV, salivary levels of citric acid, l‐glutamic acid, l‐histidine, putrescine, and cadaverine increase, indicating alterations in energy metabolism and polyamine pathways. (C) Gastrointestinal cancers exhibit characteristic metabolic changes, including increased 19‐OH‐PGE2, ARG, and lactic acid. (D) In lung cancer, N1‐Ac‐Spd emerges as a specific biomarker with diagnostic potential. (E) Breast cancer is characterized by altered amino acids, dysregulated polyamines, and lipid metabolites, highlighting the value of salivary profiling in oncology. Created in https://BioRender.com.

Metabolic profiling of saliva identifies shifts in key metabolites, including increased glucose, choline‐related compounds, and branched‐chain amino acids (BCAAs), and decreased acetate and amino acids, during the postacute phase of COVID‐19. These alterations correlate with clinical variables, reflecting inflammation, tissue repair, and metabolic disturbances, providing insights into the pathophysiology of prolonged symptoms and offering a noninvasive approach for monitoring disease progression [89] (Figure 2A). Additionally, metabolite profiling in saliva from long COVID patients identifies significant reductions in calenduloside G methyl ester, Gly Pro Lys, and creatine compared with controls. These metabolic changes correlate with diminished physical capacity and heightened fatigue, highlighting potential saliva‐based biomarkers for ongoing symptom management and pathophysiological insight into long COVID. Calenduloside G methyl ester, an oleanane‐type triterpenoid metabolite, is known for its anti‐inflammatory and immunomodulatory properties. Triterpenoids like calenduloside G methyl ester have been shown to regulate inflammatory pathways by modulating cytokine production and immune cell activity. A reduction in this metabolite in long COVID patients may contribute to persistent inflammation, potentially exacerbating the disease's pathophysiology. Additionally, calenduloside G methyl ester plays a role in cellular energy metabolism. Its reduction could impair mitochondrial function and energy production, contributing to the fatigue commonly reported in long COVID patients. While further studies are needed to confirm the exact mechanisms, the alteration of calenduloside G methyl ester levels may offer valuable insights into the metabolic disturbances and inflammatory responses associated with long COVID [90]. Similarly, saliva metabolomics reveals distinct metabolic alterations in COVID‐19 patients, with severity‐specific changes linked to disease progression. Metabolites such as sphingosine and kynurenine differentiated infected from noninfected individuals, while linoleic acid and alpha‐ketoisovaleric acid were elevated in severe cases. Salivary sphingosine and 5‐aminolevulinic acid negatively correlated with inflammatory biomarkers C‐reactive protein and D‐dimer [91]. Notably, saliva metabolomics identified a panel of six features, predominantly amino acids, capable of distinguishing high‐severity from low‐severity COVID‐19 cases with perfect diagnostic accuracy (AUC = 1.00). These findings highlight amino acid dysregulation in severe COVID‐19 and suggest saliva as a convenient, noninvasive alternative to serum for assessing disease severity and guiding timely clinical interventions [92]. Likewise, salivary metabolomic profiling using ^1^H‐NMR reveals distinct changes in metabolite levels between SARS‐CoV‐2 infected, postinfection, and noninfected individuals. Key amino acids, including alanine, glutamine, and phenylalanine, were downregulated in PCR+ patients, while metabolites such as acetate and valerate were higher in noninfected individuals. Post‐SARS‐CoV‐2 infection showed increased sucrose and butyrate levels. These findings suggest that salivary metabolomics could serve as a valuable tool for distinguishing between different stages of SARS‐CoV‐2 infection and monitoring disease progression [85]. Moreover, a combined omics approach identified two salivary metabolites, myo‐inositol (MYO) and 2‐pyrrolidineacetic acid, alongside serum protein chitinase 3‐like‐1, as effective biomarkers distinguishing COVID‐19 inpatients from outpatients. These markers correlated with microbiota shifts, including an overrepresentation of *Corynebacterium* 1 in inpatients and reduced levels of *Actinomycetaceae F0332*, *Candidatus Saccharimonas*, and *Haemophilus*. This integrative analysis highlights the potential of salivary metabolites in patient stratification and monitoring disease severity [86].

#### 5.1.2 HIV

Salivary metabolome analysis reveals significant alterations in energy metabolism and amino acid pathways among individuals with HIV, particularly post‐highly active antiretroviral therapy [93, 94]. Citric acid, l‐glutamic acid, and l‐histidine emerge as key indicators of disease progression and therapeutic response. The study also highlights interactions between oral metabolites and microorganisms, suggesting potential for using saliva as a noninvasive tool for HIV diagnostic and treatment monitoring [87] (Figure 2B). In addition, polyamine metabolism is dysregulated in the oral mucosa of HIV^+^ individuals, with elevated salivary putrescine levels correlating with altered T cell function. Increased polyamine synthesis, driven by caspase‐1, IL‐1β, and ornithine decarboxylase‐1, leads to a skewed TregDys/Th17 cell ratio. These findings suggest that saliva metabolomics can reflect immune dysfunction during chronic viral infections, offering insights into how metabolic shifts influence immune responses and T cell effector programs in HIV [95]. Moreover, salivary metabolite profiling in youth with perinatally‐acquired HIV and HIV‐exposed uninfected youth (PHEU) reveals similar overall profiles, though cadaverine levels are elevated in individuals with periodontitis, particularly among PHEU. Proteolytic processing and amino acid metabolism were prominent, highlighting the potential influence of HIV infection or treatment on oral bacterial metabolism [96] (Table 1).

**TABLE 1:** Potential saliva metabolite biomarkers for early detection/monitoring of viral diseases.

Associated virus	Methods	Saliva metabolites	Clinical applications	References
COVID‐19	^1^H‐NMR	Glucose, (CH_3_)_3_ choline‐related metabolites, taurine, acetate, creatine, histidine, lysine	Noninvasive disease monitoring	[89]
	HPLC/MS	Calenduloside G methyl ester, Gly Pro Lys, creatine	Detection for long COVID	[90]
	LC–MS/MS	Sphingosine, kynurenine, 3‐hydroxyanthranilic acid, tryptophan, 5‐aminolevulinic acid, phenylalanine, purine, alpha‐ketoisovaleric acid	COVID‐19 diagnostic and prognostic biomarkers	[91]
	LC/MS	Valine, leucine, phenylalanine, tyrosine, proline, C_44_H_74_N_8_O_16_	For disease severity assessment and clinical guidance	[92]
	^1^H‐NMR	Alanine, glutamine, phenylalanine, proline, lysine, acetate, ethanol, capronic acid, histidine, leucine	Distinction of COVID‐19 stages	[85]
	RP/UPLC–MS/MS, ESI	Myo‐inositol, 2‐pyrrolidineacetic acid	Distinguishing COVID‐19 patients	[86]
HIV	LC–MS/MS	Citric acid, l‐glutamic acid, l‐histidine, l‐asparagine, glyceric acid, adenosine, dopamine, glucosamine, deoxyguanosine, sphinganine, l‐arginine, l‐alanine, dimethylglycine	HIV diagnostic and treatment monitoring	[87]
	LC–MS, ESI–MS	Putrescine, ARG, proline, tryptophan, BCAA	Biomarker for HIV early detection	[95]
	LC–MS/MS	Cadaverine	Assessing HIV's metabolic impact on oral microbiome	[96]

### 5.2 Cancer

Saliva metabolomics is particularly effective for cancer detection due to the distinct metabolic reprogramming in tumor cells and the direct impact of cancer on the oral microenvironment. Tumors induce systemic metabolic shifts that lead to the release of specific metabolites into saliva [97, 98, 99]. Additionally, cancer progression often triggers local inflammation and immune responses, resulting in the secretion of inflammatory cytokines and metabolic byproducts into the oral cavity [100, 101, 102]. Furthermore, tumor‐induced alterations in the oral microbiome further shape the salivary metabolome, with microbial dysbiosis contributing to the production of metabolites that reflect cancerous processes [103, 104].

#### 5.2.1 Gastrointestinal Cancer

Metabolite profiling identified key salivary biomarkers, including lactic acid and kynurenic acid, capable of distinguishing gastric cancer patients from healthy controls with high diagnostic accuracy. Postoperative changes in metabolites, such as 19‐hydroxyprostaglandin E2, provide further insights into metabolic shifts following surgery. These findings underscore the potential of salivary metabolomics for noninvasive gastric cancer diagnosis and postoperative monitoring [105]. In addition, salivary diagnostic biomarkers for esophageal and gastric cancers were identified through metabolomic analyses, demonstrating comparable sensitivity to traditional serum tumor markers. Biomarkers such as cytosine for gastric cancer, and cytosine, 2‐oxoglutarate, and ARG for esophageal cancer, along with significant postoperative changes in cytosine levels, highlight the potential of saliva‐based screening for these cancers [106]. Saliva metabolite profiling combined with machine learning accurately distinguished CRC patients from healthy controls and adenoma cases. CE and LC–MS identified hydrophilic metabolites with high discriminatory power, achieving AUC values of 0.870 for CRC detection and 0.879 for CRC versus adenoma and controls [71] (Figure 2C).

Metabolomic profiling in plasma and saliva revealed distinct metabolite patterns associated with stages of liver disease, including nonalcoholic fatty liver disease (NAFLD), cirrhosis, and hepatocellular carcinoma (HCC). Two salivary metabolites including 1‐monopalmitin and 1‐monostearin specifically differentiated liver disease states, highlighting saliva's potential as a noninvasive diagnostic medium [107]. Similarly, salivary and plasma metabolite profiling identifies distinct metabolites associated with HCC, including malonic acid, which correlates between saliva and plasma. Key metabolites such as 1‐hexadecanol, succinic acid, and glycine are linked to HCC, with pathway analysis revealing dysregulation in the citric acid cycle. These findings suggest that both saliva and plasma metabolites could serve as independent biomarkers for HCC detection, highlighting the potential of salivary metabolomics in early cancer diagnosis [108]. In addition, machine‐learning analysis of salivary metabolites identified a 12‐metabolite panel, including octadecanol, lauric acid, and creatinine, that achieved 84.8% sensitivity and 92.4% specificity in detecting HCC. This panel outperformed alpha‐fetoprotein and ultrasound, suggesting salivary metabolite profiling as a noninvasive and highly accurate tool for early HCC detection, linked to liver dysfunction and disease pathology [109].

#### 5.2.2 Lung Cancer

Salivary metabolomic analysis reveals 12 key metabolites, including N1‐acetylspermidine, that differentiate lung cancer patients from controls, with significant changes observed across plasma, saliva, and tumor tissues. A machine learning model using these salivary biomarkers achieved high discriminatory power for lung cancer detection. These findings suggest that salivary metabolites, reflective of systemic and tumor‐specific changes, offer a promising noninvasive approach for early lung cancer screening [17, 99]. Additionally, salivary metabolite profiling using an ultralow noise tunable electrospray ionization liquid droplet interface MS (TELDI–MS) platform identified 23 biomarkers linked to disrupted amino acid and nucleotide metabolism in early lung cancer. Combined with transcriptomic analysis, these metabolites enabled the differentiation of early lung cancer patients from healthy controls with 97.2% sensitivity and 92% specificity [110]. Interestingly, salivary metabolite analysis identified butyrate, propionate, and hexanoate as potential predictors for radiation pneumonitis (RP) in non‐small cell lung cancer (NSCLC) patients undergoing radiotherapy. Among these, butyrate was found to be an independent risk factor for clinical RP, alongside the known factor of lung volume irradiated with >20 glycerol (Gy) [111] (Figure 2D).

#### 5.2.3 Breast Cancer

In addition to gastrointestinal cancers, saliva metabolomics has also found broad application in breast cancer [112, 113]. Salivary amino acid profiles vary significantly across molecular subtypes of breast cancer, benign breast disease, and healthy controls. Distinctive patterns in amino acids such as Asp, Gly, and Phe provide a basis for developing diagnostic models. Decision trees using multidirectional changes in amino acids like gamma‐aminobutyric acid (GABA) and Arg further delineate these groups, underscoring the importance of considering breast cancer subtypes when developing saliva‐based diagnostic markers [114]. In addition, elevated salivary polyamines, particularly spermine (SPM), were identified as potential biomarkers for breast cancer, showing strong discrimination between invasive carcinoma and healthy controls. Combining salivary metabolomics with machine learning methods, such as ADTree, achieved high diagnostic accuracy, highlighting the potential of saliva‐based noninvasive screening for breast cancer [65]. Similarly, saliva polyamine profiling, particularly the ratios of specific polyamines, offers a promising noninvasive diagnostic approach for breast cancer. Key polyamines, such as SPM, acetylspermine (Ac‐SPM), and N8‐acetylspermidine (N8‐Ac‐SPD), were found to correlate strongly with breast cancer, with a developed equation achieving an 88% concordance rate for distinguishing cancer patients from healthy individuals. The ratio of N8‐Ac‐SPD to N1‐acetylspermidine (N1‐Ac‐SPD) also serves as an index for monitoring postsurgical health, showing potential for both diagnosis and prognosis in breast cancer management [112]. Moreover, salivary metabolomics identified 18 potential biomarkers for breast cancer diagnosis, with lysophosphatidylcholine （LysoPC） (18:1), LysoPC (22:6), and monoglyceride (0:0/14:0/0:0) achieving high diagnostic accuracy [115]. Notably, salivary metabolomics identified distinct profiles in breast cancer patients, with upregulation of glycerophospholipids such as phosphatidylglycerol (PG)14:2 and specific peptides. Posttreatment reductions in these metabolites highlight their potential as biomarkers. PG14:2 demonstrated an AUC of 0.733, with 65.22% sensitivity and 77.14% specificity, suggesting its utility for noninvasive cancer monitoring [113]. Interestingly, salivary volatile profiling using HS–SPME/GC–MS identified distinct biosignatures for breast cancer across geographically distant populations. Key volatiles, including 3‐methyl‐pentanoic acid, phenol, acetic acid, and decanal, effectively differentiated breast cancer patients from controls [116] (Figure 2E).

#### 5.2.4 Other Cancer Types

Salivary and plasma metabolite profiling in glioblastoma patients revealed distinct metabolic signatures linked to clinical outcomes. Elevated levels of cyclic‐adenosine monophosphate, 3‐hydroxy‐kynurenine, and dihydroorotate were associated with unfavorable progression‐free survival, affecting key pathways like the pentose phosphate and Warburg effect. The lipid profiles of patients with unfavorable outcomes showed greater heterogeneity and fewer marker associations compared with those with favorable outcomes [117]. Additionally, amino acid profiling in saliva reveals significant differences in alanine, valine, proline, and phenylalanine concentrations between papillary thyroid carcinoma (PTC) patients and healthy controls. A diagnostic panel combining these metabolites achieved high accuracy for early PTC detection [118] (Table 2).

**TABLE 2:** Potential saliva metabolite biomarkers for early detection/monitoring of cancers.

Cancers	Methods	Saliva metabolites	Clinical applications	References
Gastrointestinal cancer	LC–MS	Lactic acid, kynurenic acid, 3‐hydroxystachydrine, S‐(1,2,2‐trichlorovinyl)‐l‐cysteine, 19‐hydroxyprostaglandin E_2_	For gastric cancer diagnosis and monitoring	[105]
	LC–MS	Cytosine, 2‐oxoglutarate, ARG	Saliva‐based screening for gastric	[106]
	CE–MS LC–MS	Lactate, pyruvate, N^1^‐acetylspermine, N^1^, N^8^‐diacetylspermidine	Clinical screening for adenoma and CRC	[71]
	GC–TOF MS	1‐Monopalmitin, 1‐monostearin	Diagnostic of NAFLD, cirrhosis, HCC	[107]
	GC–TOF MS	1‐Hexadecanol, isooctanol, malonic acid, N‐acetyl‐valine, succinic acid	Biomarkers for HCC detection	[108]
	GC–TOF MS	Octadecanol, acetophenone, lauric acid, l‐monopalmitin, dodecanol, salicylaldehyde, glycyl‐proline, l‐monostearin, creatinine, glutamine	Biomarkers for early HCC detection	[109]
Breast cancer	LC–MS	Asp, Gly, Leu+Ile, Orn, Phe, Pro, Thr, Tyr, GABA, Hyl, Arg, His, Pro	Distinction molecular subtypes of breast cancer	[114]
	CE–TOFMS LC–QQQMS	SPM, N_1_‐acetylspermine, ribulose‐5‐phosphate	Screening for breast cancer	[65]
	UPLC–ESI–MS/MS	CAD, SPM, SPD, Ac‐SPM, N1‐Ac‐SPD, N8‐Ac‐SPD	Biomarkers for breast cancer diagnosis and prognosis	[112]
	HILIC, RPLC UPLC–ESI–MS	Palmitic amide, phytosphingosine, acetylphenylalanine, propionylcholine, phenylalanine, citrulline, histidine, N‐acetylneuraminic acid, 4‐hydroxyphenylpyruvic acid	Biomarkers for breast cancer diagnosis	[115]
	LC–Q‐TOF/MS	Donazepil, dioscin, dilazep, tetrahydrogambogic acid	Noninvasive cancer monitoring biomarkers	[113]
	HS–SPME/GC–MS	3‐Methyl‐pentanoic acid, phenol, acetic acid, decanal, 4‐methyl‐pentanoic acid, p‐tert‐butyl‐phenol, propanoic, benzoic acids, 1,2‐decanediol, 2‐decanone	For breast cancer diagnosis	[116]
Lung cancer	CE–TOF–MS QQQ–MS	N^1^, N^8^‐diacetylspermidine, N^1^‐acetylspermidine, N^8^‐acetylspermidine, SPM, phenylalanine, histidine, tryptophan, 2‐aminobutyric acid, succinate, 5‐oxoproline	Biomarkers for lung cancer detection	[17]
	TELDI–MS	GABA, cytosine, uracil, creatinine, pyroglutamic acid, ketoleucine, adenine, allysine, guanine, N‐acetylproline, N‐acetylhistidine, serine, proline, valine, xanthine, ARG	Noninvasive screening for lung cancer	[110]
	CE–TOFMS LC–QQQMS	Butyrate, propionate, hexanoate	Potential predictors for RP in NSCLC	[111]
Glioblastoma	LC–QqQ–MS LC–QTOF–MS	Indoline‐2‐carboxylate, cytosine, 2‐ketobutyrate, adenosine 3‐5‐cyclic monophosphate, 3‐hydroxy‐dl‐kynurenine, l‐dihydroorotic acid, dl‐valine, 4‐hydroxybenzoic acid	Prognostic biomarkers for glioblastoma	[117]
Thyroid cancer	HILIC–UPLC–HRMS	l‐Alanine, l‐methionine, l‐phenylalanine, l‐tryptophan, l‐proline, l‐threonine, l‐leucine, l‐valine, l‐glycine, l‐isoleucine	Biomarkers for PTC early diagnosis	[118]

### 5.3 Sjögren's Syndrome

Saliva metabolomics is particularly well suited for studying SS due to the unique metabolic alterations associated with the dysfunction of salivary glands and systemic autoimmune processes [119, 120]. In SS, the immune system targets exocrine glands, including the salivary and lacrimal glands, leading to chronic inflammation, reduced salivary secretion, and altered glandular function. This inflammation triggers metabolic shifts, which can be detected in saliva [121, 122]. Moreover, the systemic nature of the disease, involving widespread immune activation and dysregulated cytokine production, further influences salivary composition. Saliva metabolomics can capture these systemic immune responses, providing a snapshot of both local glandular dysfunction and systemic inflammation.

**FIGURE 3:**
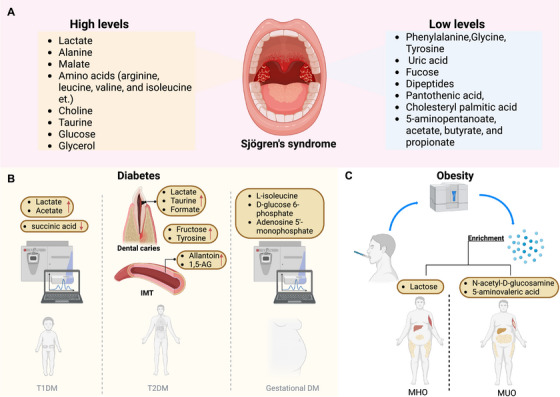
Salivary metabolic markers in systemic diseases. (A) In SS, salivary metabolomics reveals increased or decreased levels of lactate, amino acids, and related metabolites, reflecting glandular dysfunction. (B) In diabetes, succinate levels are reduced while lactate and acetate are increased in children with type 1 diabetes, whereas multiple metabolite concentrations are elevated in type 2 diabetes. Additional markers, including lactate, taurine, and formate, correlate with higher dental caries risk, while 1,5‐anhydroglucitol (1,5‐AG) is identified as a strong predictor of IMT. (C) Lactose, N‐acetyl‐d‐glucosamine, and 5‐aminovaleric acid can distinguish MHO from MUO individuals, highlighting diagnostic potential in metabolic disorders. Created in https://BioRender.com.

Targeted metabolomics of saliva from SS patients reveals elevated concentrations of lactate, alanine, malate, and amino acids like ARG, leucine, valine, and isoleucine, associated with oxidative stress and T‐cell proliferation. This differentiation suggests that saliva can effectively mirror the cellular changes in the microenvironments of affected salivary glands, offering a noninvasive diagnostic tool for SS [123]. Similarly, salivary metabolomic profiling identifies 91 differentially expressed metabolites in SS patients, with 16 confirmed identifications, including alanine, isovaleric acid, and succinic acid. These metabolites, linked to amino acid and purine metabolism, exhibit strong sensitivity and specificity, with alanine and isovaleric acid showing perfect sensitivity for SS detection [124]. Notably, metabolic profiling revealed significant regulation of tryptophan, tyrosine, carbon fixation, and aspartate‐asparagine pathways in primary SS (pSS), linked to inflammation, cognitive impairment, and immune response. Phenylalanyl‐alanine demonstrated strong predictive ability for pSS, with an AUC of 0.87 in the testing group and 0.75 in validation, highlighting its potential as a biomarker for differential diagnosis [125]. In addition, salivary metabolite analysis in pSS reveals consistently elevated levels of choline, taurine, alanine, and glycine compared with controls, despite significant inter‐ and intra‐individual variations. Choline demonstrated the lowest intrapatient variability, highlighting its potential as a stable biomarker for pSS monitoring [126]. Salivary metabolomics reveals a distinct metabolic profile in pSS patients, characterized by reduced levels of glycine, tyrosine, uric acid, and fucose, reflecting salivary gland destruction. Principal component analysis (PCA) demonstrated decreased metabolite diversity in pSS samples compared with healthy controls, with two subpopulations identified based on metabolite profiles [120]. Moreover, metabolomic analysis revealed altered salivary profiles in pSS, with reduced levels of metabolites such as dipeptides, phenylalanine, pantothenic acid, and cholesteryl palmitic acid compared with healthy controls. Low‐dose doxycycline treatment normalized several metabolites linked to oral microbiota dysbiosis in pSS patients. These findings underscore the potential of salivary metabolite profiles in understanding and monitoring pSS pathology and treatment responses [127]. Furthermore, salivary metabolomic profiling using NMR spectroscopy identifies significant metabolic alterations in pSS patients, including increased levels of glucose, glycerol, taurine, and lactate, and decreased levels of 5‐aminopentanoate, acetate, butyrate, and propionate. Additionally, choline and fucose levels show age‐specific and population‐specific changes [128]. Likewise, salivary metabolomic profiling using ^1^H‐NMR reveals distinct metabolic differences between pSS patients and healthy controls. Unsupervised PCA and partial least squares (PLSs) discriminant analysis identified a unique set of metabolites that differentiate pSS from healthy individuals, highlighting the potential of salivary metabolomics as a tool for understanding pSS pathology [129] (Figure 3A).

### 5.4 Diabetes

Diabetes is a chronic metabolic disorder characterized by high blood sugar levels due to the body's inability to produce sufficient insulin (type 1 diabetes [T1DM]) or to effectively use the insulin it produces (type 2 diabetes [T2DM]) [130, 131]. Accumulative evidence has suggested that saliva metabolomics is effective for early detection, diagnosis, and monitoring of diabetes. For instance, salivary metabolomic scores, primarily linked to fructose, tyrosine, and amino acid metabolism, predict incident prediabetes, while plasma and multifluid metabolomic scores are stronger predictors of T2DM. Higher saliva metabolomic scores correlate with an increased risk of prediabetes, and all three metabolomic scores are associated with a lower likelihood of regressing from prediabetes to normoglycemia. These findings highlight the potential of saliva‐based biomarkers in early diabetic risk assessment [132]. Additionally, salivary metabolite profiling using NMR revealed distinct differences between healthy children and those with uncontrolled T1DM, with key metabolites such as lactate, acetate, and sucrose contributing to group separation. Metabolites like succinic acid were decreased, while lactate and acetate were elevated in T1DM children with poor glycemic control [133]. Similarly, metabolic profiling revealed distinct salivary metabolite signatures between well‐controlled and poorly controlled diabetes based on hemoglobin A1c levels. Principal component and orthogonal PLSs analyses demonstrated clear separation of metabolic profiles in both T1DM and T2DM. These findings highlight the potential of salivary metabolites as noninvasive biomarkers for monitoring glycemic control and disease management in diabetes [134]. Notably, untargeted metabolomic analysis of saliva revealed distinct metabolic profiles in pregnant women with gestational diabetes mellitus (DM), healthy pregnant women, and healthy nonpregnant women. Nine differential metabolites, including l‐isoleucine, d‐glucose 6‐phosphate, and adenosine 5′‐monophosphate, were identified with high confidence, enriching pathways linked to metabolic dysregulation [6]. Interestingly, saliva metabolomics is increasingly recognized for its ability to uncover the risk of diabetes‐related complications, offering a window into the broader impact of metabolic dysregulation in DM. Salivary metabolomics reveals distinctive biomarkers in individuals with T2DM, including elevated levels of lactate, taurine, and formate, which correlate with increased dental caries risk. This differentiation, demonstrated through ^1^H‐NMR analysis, underscores the potential of salivary metabolites in identifying diabetes‐related oral health complications and highlights the link between metabolic changes and dental caries pathogenesis in T2DM patients [135]. Similarly, salivary metabolomics revealed that metabolites like allantoin and 1,5‐anhydroglucitol (1,5‐AG) are strong predictors of carotid intima‐media thickness (IMT) in T2DM patients. Despite treatment‐induced changes in metabolite levels, the relationship between these metabolites and IMT remained consistent, underscoring the potential of salivary metabolomics as a noninvasive tool for assessing atherosclerotic risk in T2DM [136]. Moreover, in the San Juan overweight adult longitudinal study, metabolomic profiling of saliva and plasma did not predict T2DM progression but was linked to specific cardiometabolic biomarkers. Metabolites related to lysine and pyrimidine metabolism were identified, with significant associations observed between metabolomic signatures and increases in low‐density lipoprotein, insulin resistance, and triglyceride levels after 3 years [137] (Figure 3B).

### 5.5 Obesity

Salivary metabolomics has emerged as a promising tool for identifying biomarkers of metabolic health in obese individuals [137]. For instance, tandem ionization GC×GC–TOF MS revealed distinct salivary metabolomic signatures differentiating metabolically healthy obese (MHO) from metabolically unhealthy obese (MUO) individuals. These findings highlight potential biomarkers linked to metabolic health in obesity [138]. Similarly, distinct salivary metabolomic signatures were identified in pediatric obesity, NAFLD, and metabolic syndrome, with notable differences in metabolites related to energy metabolism, amino and organic acids, and gut microbiota activity. These findings suggest a role for salivary metabolites in reflecting disease pathogenesis and may guide targeted monitoring and treatment strategies [139] (Figure 3C).

### 5.6 Neurodegenerative Disorder

#### 5.6.1 Alzheimer's Disease

AD is a progressive neurodegenerative disorder characterized by the gradual deterioration of cognitive functions, primarily affecting memory, executive function, and language [140, 141]. It is marked by the accumulation of amyloid‐beta plaques and neurofibrillary tangles of tau protein in the brain, leading to neuronal loss and brain atrophy [142, 143]. Saliva analysis in AD and mild cognitive impairment (MCI) revealed significant alterations in metabolites and proteins linked to multiple cellular pathways. MS‐based metabolomics and proteomics identified systemic metabolic disruptions correlating with disease progression. These findings highlight the potential of salivary biomarkers for distinguishing AD, MCI, and healthy states through multiomics integration [144]. Similarly, salivary metabolomics identified distinct biomarker panels capable of differentiating AD, MCI, and cognitively normal individuals with high diagnostic accuracy (AUC up to 1.000). Using isotope labeling and LC–MS, 6230 metabolites were profiled, revealing potential diagnostic metabolites and panels for AD progression [145]. Additionally, salivary metabolomics revealed significantly reduced acyl‐alkyl phosphatidylcholines in AD and MCI patients, highlighting specific lipid alterations [146]. Notably, altered periodontal status in AD patients is associated with significant changes in salivary metabolism. Higher plaque index and bleeding on probing were observed in AD patients. Candidate biomarkers, including Cis‐3‐(1‐carboxy‐ethyl)‐3,5‐cyclohexadiene‐1,2‐diol and dodecanoic acid, were identified using metabolomic analysis. Oral microbiota dysbiosis was linked to these metabolic shifts, suggesting that changes in bacterial flora contribute to metabolic alterations in AD [147]. Interestingly, saliva metabolomics using NMR spectroscopy revealed distinct metabolic profiles between dementia patients and controls, with increased concentrations of acetic acid, histamine, and propionate, and decreased levels of dimethyl sulfone, glycerol, taurine, and succinate. These metabolites, including histamine and taurine, are linked to AD, while dietary and microbiota‐derived metabolites, such as dimethyl sulfone and propionate, may reflect periodontal health. The findings suggest the potential of saliva as a noninvasive tool for early screening and detection of dementia [148].

#### 5.6.2 Parkinson's Disease

PD is a chronic and progressive neurodegenerative disorder characterized by the loss of dopamine‐producing neurons in the substantia nigra [149, 150, 151]. Salivary metabolomic profiling in PD revealed elevated levels of metabolites such as phenylalanine, glycine, taurine, and N‐acetylglutamate, implicating disruptions in amino acid, energy, and neurotransmitter metabolism. Early‐stage PD showed higher metabolite concentrations than advanced PD, potentially linked to dopaminergic treatment. These findings highlight metabolic imbalances involving gut microflora and energy pathways, contributing to PD pathogenesis [152].

### 5.7 Sleep Disorder

Saliva metabolite analysis in obstructive sleep apnea (OSA) revealed three key metabolites—peroxidized hydroxy‐octadecenoic acid phosphatidylcholine (PHOOA‐PC), keto‐peroxidized octadecenoic acid phosphatidylcholine, and 9‐nitrooleate—upregulated in postsleep samples from OSA patients. PHOOA‐PC was positively correlated with the apnea‐hypopnea index and inversely related to salivary surface tension. Metabolite set enrichment analysis highlighted arachidonic acid metabolism as significantly upregulated in OSA [153]. Moreover, alterations in the oral microbiome and salivary metabolites were observed in OSA patients with comorbid hypertension (HTN). Genera such as *Haemophilus*, *Neisseria*, and *Oribacterium* were enriched in these patients, while *Actinomyces* abundance was reduced. Metabolomic analysis revealed increased 2‐hydroxyadenine levels in OSA patients and decreased L‐carnitine in those with OSA and HTN. These findings highlight potential noninvasive biomarkers and provide insights into the relationship between oral microbiota, salivary metabolites, and cardiovascular disease in OSA‐associated HTN [154].

### 5.8 Gastrointestinal Diseases

Salivary metabolomics identified altered levels of propionic acid, putrescine, tyrosine, and other metabolites in chronic hepatitis B, implicating disruptions across nine metabolic pathways. These findings highlight the potential of salivary metabolomics for noninvasive diagnosis and improved understanding of hepatitis B pathophysiology [84]. In addition, differentially expressed salivary metabolites, including 12‐hydroxydodecanoic acid, hypoxanthine, and inosine, were identified as potential biomarkers for drug‐induced liver injury (DILI) in a cohort of patients and healthy controls. Using untargeted metabolomics, a set of eight metabolites closely associated with liver injury showed promising diagnostic potential, with area under the curve values ranging from 0.733 to 1 [155].

### 5.9 Other Systemic Disorders

**FIGURE 4:**
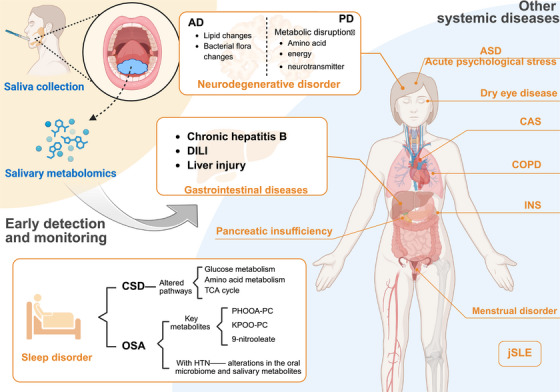
Diagnostic and monitoring applications of salivary metabolomics in systemic diseases. Salivary metabolomics enables detection of metabolic alterations in a broad spectrum of systemic disorders. In neurodegenerative diseases such as AD and PD, metabolite shifts reflect neuronal dysfunction. Gastrointestinal diseases including chronic hepatitis B and DILI show characteristic metabolic perturbations. Sleep disorders such as CSD and OSA are also associated with distinctive salivary signatures. Furthermore, salivary profiling has been applied to evaluate acute psychological stress, dry eye disease, menstrual disorders, and jSLE. Noninvasive collection makes it a practical approach for early diagnosis, longitudinal disease monitoring, and therapeutic evaluation. Created in https://BioRender.com.

Increasing evidence has shown that saliva metabolomics is effective in the diagnosis and management of various systemic disorders [156]. For instance, lipidomics analysis identified 22 lipid‐based cannabis‐responsive biomarkers in children with autism spectrum disorder undergoing medical cannabis (MC) treatment, showing a shift toward levels observed in typically developing children. Changes in sphingomyelin and associations with inflammation, oxidative stress, and neuron function suggest a role for MC in modulating lipid metabolism and neuroinflammation [157]. In addition, saliva metabolomics reveals alterations in lipid metabolism associated with cystic fibrosis, a genetic disorder affecting chloride ion transport. These metabolic disruptions correlate with disease manifestations, including chronic obstructive pulmonary disease, *Pseudomonas Aeruginosa* infection, pancreatic insufficiency, liver dysfunction, and diabetes‐related complications. Salivary analysis in children with idiopathic nephrotic syndrome reveals significant dysbiosis in the oral microbiota, with increased *Actinobacteria* and *Firmicutes*, and decreased *Bacteroidota* and *Proteobacteria*. Metabolic profiling identifies 146 differentially expressed metabolites, linked to pathways such as ascorbate metabolism and terpenoid biosynthesis [158]. Moreover, metabolomic analysis of tears and saliva from patients with dry eye disease reveals altered metabolite profiles, with 56 significantly different metabolites in tear samples linked to oxidative stress and the ocular microbiome. In saliva, lower levels of hypotaurine were observed in patients with tear film instability [159]. Notably, salivary metabolomics in adolescents with juvenile systemic lupus erythematosus (jSLE) revealed a distinct metabolic profile, with elevated N‐acetyl sugars and reduced levels of phenylalanine, glycine, taurine, hydroxybutyrate, and valerate compared with healthy controls [16]. Salivary analysis in patients with carotid atherosclerosis (CAS) and periodontitis reveals distinct microbial profiles and metabolic pathways. Increased abundance of *Streptococcus*, *Lactobacillus*, and Cutibacterium, alongside elevated levels of trimethylamine N‐oxide and leukotriene D4, correlate with CAS. Concurrently, reductions in carnosine and the presence of periodontal pathogens suggest a linkage between oral health and atherosclerosis progression, indicating the potential of saliva as a biomarker source for cardiovascular conditions [160]. Interestingly, acute psychological stress induces significant changes in the salivary metabolome, including decreases in amino acids, B‐vitamins, and metabolites involved in B‐vitamin‐dependent reactions. These alterations are linked to the pathology of psychological and neurodegenerative disorders, suggesting a potential neuroprotective role. Salivary metabolomic profiling provides a molecular fingerprint of stress, highlighting candidate biomarkers for stress‐related diseases [161]. Notably, saliva metabolomics reveals altered metabolic profiles in women with menstrual disorders caused by high‐temperature environments. Key pathways involve carbohydrate metabolism, membrane transport, and nucleotide metabolism, with metabolites like N‐acetylneuraminic acid and MYO linked to disruptions [162] (Figure 4).

Despite the variability in salivary metabolite profiles reported across different studies of the same disease, emerging patterns of consistency suggest shared underlying metabolic disruptions that warrant further investigation. Such variability is common in the early stages of biomarker discovery, especially in complex diseases like cancer, where differences in study design, patient demographics, and disease stages can contribute to divergent findings. However, the repeated identification of certain metabolites within specific disease categories points to potentially universal metabolic alterations with diagnostic relevance.

For instance, in liver diseases such as HCC, cirrhosis, primary biliary cholangitis (PBC), and DILI, recurring salivary metabolites, including fatty alcohols and metabolites related to mitochondrial function and energy metabolism, have been consistently identified. The presence of fatty alcohols—such as 1‐hexadecanol, isooctanol, and 12‐hydroxydodecanoic acid—suggests dysregulation in lipid metabolism, specifically ω‐oxidation and peroxisomal fatty acid degradation [107, 155]. These alterations may reflect impaired hepatic clearance or disrupted systemic lipid homeostasis. Similarly, saliva metabolite profiles from both HCC and PBC patients revealed changes in metabolites related to mitochondrial and energy metabolism. In HCC, several salivary metabolites including malonic acid and succinic acid—both directly involved in the tricarboxylic acid cycle—were significantly elevated, while in PBC, the enrichment of carboxylic acids such as glutaric acid, succinate, and fumaric acid was also closely associated with liver dysfunction, indicating sustained disruption of mitochondrial pathways [108, 163]. These metabolic shifts likely reflect a common feature of chronic liver injury: mitochondrial dysfunction and reprogrammed energy metabolism, which occur in response to sustained oxidative stress, inflammation, and altered hepatocellular bioenergetics. These recurring metabolite patterns across various liver diseases underscore their potential as universal biomarkers of liver dysfunction, reinforcing the value of salivary metabolomics as a noninvasive tool for early diagnosis and disease monitoring [10].

In malignancies, including breast, oral, and pancreatic cancers, elevated levels of amino acids—such as BCAAs (leucine, isoleucine, valine), phenylalanine, proline, glycine, and alanine—have also been consistently observed [113, 118, 164]. These changes in amino acid concentrations reflect metabolic adaptations in rapidly proliferating tumors, supporting biosynthetic demands, redox control, and extracellular matrix remodeling. The recurrence of these metabolites across different cancer types further strengthens their potential as generalizable salivary biomarkers of tumor metabolism.

In conclusion, despite the variability observed in metabolite profiles across studies, the consistent identification of certain metabolites across different disease categories provides valuable insight into shared metabolic pathways. These findings highlight the potential of salivary metabolomics as a reliable, noninvasive diagnostic platform for early detection and monitoring of systemic diseases, including liver diseases and cancers, with implications for personalized treatment strategies (Table 3).

**TABLE 3:** Clinical trials on salivary metabolomics and related diseases are currently underway.

Biomarker	Sponsor	Aiming	Status	Study starts	NCT number
N/A	Mayo Clinic	Study explores HPV biomarkers in saliva for cancer stage	Completed	2021‐09‐17	NCT05093400
NLRP3	University of Messina	Exploring the connection between periodontitis and diabetes	Completed	2014‐06‐01	NCT04450810
Estrogen and progesterone	Bezmialem Vakif University	Improving gum problem detection and prevention during pregnancy	Recruiting	2025‐06‐24	NCT07054177
Neuropeptides, hormones, inflammatory cytokines	University Hospital, Tours	Analyzing salivary metabolomics in BMS patients and healthy controls	Completed	2021‐03‐10	NCT04704128
Malondialdehyde	University of Messina	Revealing the link between periodontitis and CHD	Completed	2014‐01‐03	NCT04030286
ARG	University of Messina	Revealing the link between periodontitis and CHD	Completed	2014‐01‐06	NCT03873948
N/A	Fondazione Don Carlo Gnocchi Onlus	Optimizing the onset of ALS and stratification of ALS patients	Completed	2019‐07‐01	NCT04233099
N/A	West Virginia School of Osteopathic Medicine	Assessing salivary biomarkers for risk stratification in metabolic syndrome	Completed	2010‐03‐11	NCT01086137
N/A	University of Sao Paulo	Monitoring periodontal condition of patients with type 1 diabetes	Completed	2013‐11	NCT02935868

This table was obtained from the https://clinicaltrials.gov/ website for the purpose of this study.

## 6 Key Factors and Optimization Toward Precision Salivary Metabolomics

Accurate and reproducible salivary metabolomics relies on a thorough understanding of the factors that influence the composition and dynamics of saliva. Numerous variables, including sampling methodology, storage conditions, sampling time, age, and exogenous stimuli, significantly affect the metabolic profile of saliva. For instance, salivary composition varies spatially and temporally across the oral cavity, with distinct metabolite profiles and diurnal patterns linked to different salivary subtypes [165]. Comprehensive metabolomic profiling reveals location‐specific metabolites and novel oscillating patterns, highlighting the dynamic and functional complexity of the salivary metabolome. Optimizing protocols for sample collection, storage, and analysis, while accounting for these variables, is essential to ensure reliable and meaningful results in salivary metabolomics. A standardized approach that addresses these factors will not only improve reproducibility but also enhance the potential for salivary metabolomics in clinical diagnostics and disease biomarker discovery [166].

### 6.1 Age

Salivary metabolite profiles vary with age, salivary gland maturation, and dentition in infants and young children. Distinct metabolic changes were observed before and after 30 months of age, with higher levels of alanine, choline, and lactate in younger children, and increased levels of acetate, butyrate, leucine, and succinate in older children. These findings highlight the influence of age and dental eruption on the salivary metabolome [167]. Similarly, longitudinal analysis of infant saliva reveals significant changes in 18 metabolites during the first months of life, largely associated with increased oral microbial metabolism. These metabolic shifts occur independently of milk feeding history, highlighting the dynamic nature of the salivary metabolome during early development [168]. In addition, underwater exercise in older adults led to significant reductions in salivary amino acids, including glycine and proline, and bacterial counts, while halitosis and oxidative stress markers remained unchanged. Older individuals exhibited higher baseline levels of 166 metabolites compared with younger counterparts, with 15 metabolites significantly reduced during exercise [169].

### 6.2 Sample Collection and Handling

#### 6.2.1 Sampling Methodology

Sampling methods significantly influence the analysis of salivary metabolites, with spitting and aspiration providing comparable results for most metabolites, while the Salivette method yielded inconsistent quantifications. Precollection oral rinsing has been shown to reduce salivary ammonia concentration, while filtration of saliva samples can mitigate degradation‐related artifacts, underscoring the importance of preanalytical control in obtaining consistent and interpretable metabolic readouts [170]. Mouth rinsing affected metabolite concentrations and introduced variability, suggesting nonrinsing methods as preferable for untargeted metabolomic studies. Similarly, saliva, collected via various methods, exhibits distinct metabolic profiles, emphasizing the need for standardized collection protocols in metabolomic studies [171]. In addition, different extraction methods and solvents for salivary metabolites yield varying results, with protein precipitation (PPT) offering the highest metabolite coverage. Among PPT solvents, acetonitrile provided the best repeatability, while acetone achieved the highest signal intensities. Liquid–liquid extraction with chloroform and methanol was optimal for extracting small hydrophobic compounds. These findings guide the selection of extraction techniques for targeted and untargeted salivary metabolomics analysis [172]. An optimized protocol for ^1^H‐NMR metabolic profiling enables precise identification and quantification of low‐concentration metabolites across whole, parotid, and submandibular/sublingual saliva subtypes. This approach enhances the reproducibility of salivary metabolomic analyses, supporting its use in uncovering biomarkers and mechanisms in various human diseases [173]. CE time‐of‐flight MS (CE–TOFMS) enables comprehensive profiling of charged salivary metabolites, facilitating biomarker discovery for both oral and systemic diseases. Optimized sample pretreatment protocols improve migration time reproducibility, enhance peak alignment, and ensure consistent metabolite quantification, advancing untargeted metabolomic analysis for identifying novel diagnostic biomarkers [174].

#### 6.2.2 Sample Storage

Salivary metabolite stability varies with storage and preparation conditions. At −20°C, samples remain stable for at least 4 weeks, while room temperature or 4°C storage induces metabolic shifts within 6–48 h, including decreases in lactate, alanine, and choline, and increases in acetate. Preparation with NaN_3_ prevents early changes in fucose, proline, and xylose. These findings emphasize optimized protocols to preserve salivary metabolites [31]. Likewise, storage at room temperature increased amino acids and decreased other metabolites, with minimal changes observed at −20°C over 30 days [175, 176].

#### 6.2.3 Sampling Time

Sampling time, particularly morning hours, significantly influences salivary metabolite profiles more than sleep deprivation itself, as revealed by semi‐targeted metabolomics in healthy young males. Variations in metabolites were notably more pronounced due to the time of sampling rather than the effects of a single night's sleep loss. This underscores the necessity of controlling for sampling time and sleep–wake history in oral fluid metabolomics research to avoid misinterpretations of metabolic data [177]. Additionally, CE–MS of mouth‐rinsed water from healthy participants reveals that 100 out of 167 frequently detected metabolites exhibit significant intraday variations, potentially influenced by meals, oral hygiene, or circadian rhythms. No interday variations were observed. These findings inform the optimal timing for collecting mouth‐rinsed samples in clinical research, enhancing metabolome analysis accuracy [178]. Moreover, diurnal variations significantly influence salivary metabolomic profiles, particularly in stimulated saliva, while secretion volume remains consistent across inter‐ and intraday assessments. These findings underscore the necessity of standardized sampling times to minimize bias in clinical applications of salivary metabolomics [179, 180].

### 6.3 Diet and Lifestyle Influence

Emerging evidence underscores the significant influence of diet and lifestyle on the salivary metabolome, shaping its composition through both systemic and local mechanisms [181]. Comparative multiomics analyses have demonstrated that individuals following distinct dietary patterns—such as vegan, seafood‐based omnivore, and red meat‐based omnivore diets—exhibit discernible differences in their salivary metabolomic profiles [182, 183]. For instance, nontargeted metabolomic profiling has identified heightened activity in lipid, amino acid, and nucleotide metabolism pathways among vegetarians, with machine learning models successfully classifying individuals based on specific salivary metabolites such as histidinyl‐valine [184]. Similarly, diet‐related metabolite markers, including formate, urea, 1‐propanol, and hexanoic acid, have been found to distinguish omnivores, ovo‐lacto‐vegetarians, and vegans, even in the absence of significant shifts in salivary microbial composition [185].

Further studies have shown that adherence to a Mediterranean dietary pattern is linked to favorable salivary metabolic traits, such as an increased basal metabolic rate and the enrichment of microbial taxa like *Prevotella* and *Subflava*, which correlate with macronutrient metabolism and respiratory quotient [181]. In addition to dietary patterns, lifestyle factors, such as long‐term mastication, also impact salivary metabolite profiles. For example, chewing interventions have led to significant changes in over 40 metabolic features, particularly those related to glycogenic amino acid and energy metabolism [186]. These findings emphasize the necessity of considering both dietary and lifestyle factors when interpreting salivary metabolomic data. Unaccounted‐for variations in these factors may confound biomarker discovery and reduce reproducibility across studies, particularly those aimed at detecting systemic disease signatures in saliva.

### 6.4 Exogenous Stimuli

**FIGURE 5:**
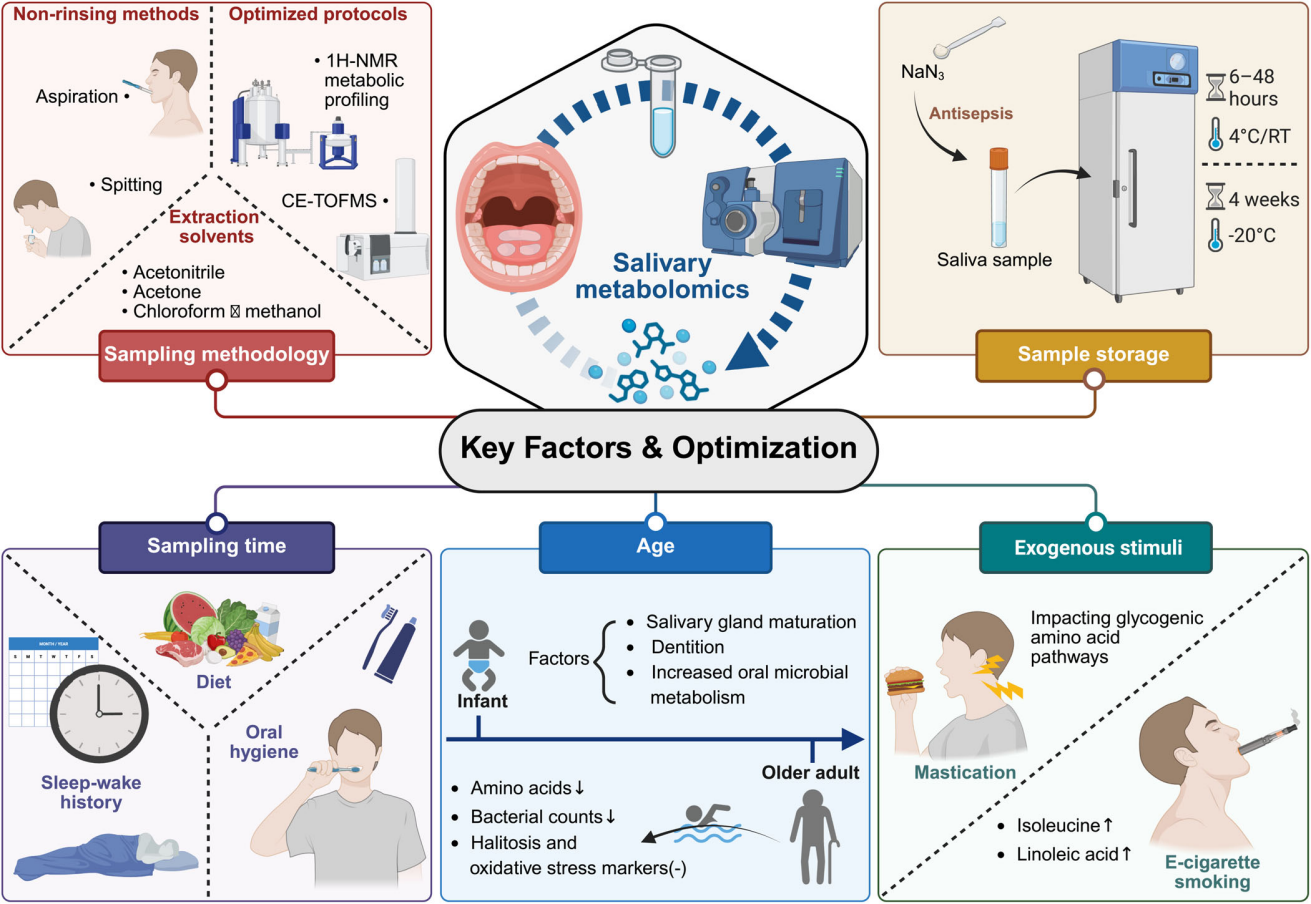
Key factors and optimization strategies for reliable salivary metabolomics. Critical determinants of reliable salivary metabolomics include sampling methods, storage conditions, timing, age, and exogenous factors. Standardized collection approaches, such as spitting or aspiration, combined with analytical protocols including ^−^H‐NMR and CE–TOFMS, improve reproducibility. Appropriate preservation with sodium azide and freezing ensures metabolite stability. Sampling time strongly influences profiles due to circadian rhythms, diet, and oral hygiene. Age‐related differences are observed across life stages, from gland maturation and dentition in infants to exercise‐induced metabolic changes in older adults. Exogenous stimuli, including mastication and e‐cigarette use, affect metabolic pathways, particularly those linked to glycogenic amino acids and inflammation. Careful optimization of these factors enhances diagnostic reliability and clinical applicability. Created in https://BioRender.com.

Exogenous stimuli, such as physical and mechanical factors, can significantly influence the composition of metabolites in saliva. long‐term mastication induces significant changes in salivary metabolite profiles, with 41 metabolites, including alanine, ARG, and glutamine, showing altered concentrations. Most metabolites decreased following the intervention, except for a few such as citrate. Pathways related to glycogenic amino acids were notably affected, highlighting the influence of physical stimuli like mastication on salivary metabolic composition and its potential implications for oral and systemic health monitoring [187]. Similarly, in children aged 3–12 years of age, mechanical stimulation increases salivary flow and alters metabolomic profiles, with stimulated saliva showing higher levels of isoleucine, N‐acetyl sugar, and other metabolites compared with unstimulated samples [188]. Saliva analysis from e‐cigarette users reveals significant metabolic alterations, including elevated levels of linoleic acid, elaidic acid, and glucuronic acid—metabolites that are mechanistically linked to inflammation and detoxification. Increased linoleic acid promotes the synthesis of arachidonic acid‐derived prostaglandins and leukotrienes, molecules known to drive systemic inflammation, potentially contributing to cardiovascular diseases [189, 190, 191]. Elaidic acid, a trans‐fatty acid, has been shown to activate the NLRP3 inflammasome through endoplasmic reticulum stress and MAPK signaling pathways, intensifying the release of proinflammatory cytokines, further exacerbating inflammation [192]. Additionally, elevated glucuronic acid reflects heightened hepatic glucuronidation in response to e‐cigarette flavorants, a process associated with oxidative stress and inflammatory signaling pathways. This increase in oxidative stress may impair endothelial function and contribute to vascular inflammation. These molecular changes are supported by clinical markers of compromised health, including elevated exhaled carbon monoxide and reduced oximetry readings, indicating an overall increase in inflammatory and oxidative burden [193]. Collectively, these findings underscore the potential of saliva as a sensitive, noninvasive marker of the inflammatory and oxidative effects of e‐cigarette use [189] (Figure 5).

## 7 Challenges

Salivary metabolomics offers significant potential for noninvasive, early diagnosis and monitoring of systemic diseases. However, despite its promise, the application of salivary metabolomics in clinical practice faces considerable challenges. The complexity and variability in salivary metabolite profiles pose substantial challenges in identifying precise biomarkers for early detection or prognostic assessment of systemic conditions [76, 166]. These inherent sources of variation make it difficult to pinpoint metabolites that are consistently and reliably associated with specific diseases. Additionally, the dynamic nature of the salivary metabolome, influenced by both biological and environmental factors, requires robust methodologies to account for these variations while ensuring accuracy and reproducibility in clinical settings [194].

To address these challenges, two key strategies must be prioritized. One critical approach is enhancing the sensitivity and accuracy of metabolomics technologies. Advanced analytical platforms can be optimized to detect low‐concentration metabolites and improve quantitative precision. Employing cutting‐edge data processing techniques will further refine the identification of relevant biomarkers, allowing for more accurate and sensitive detection of disease‐related metabolic alterations [65, 71, 195]. For instance, a data processing strategy for two‐dimensional GC with time‐of‐flight MS was optimized to address misalignments and response fluctuations in salivary metabolomic analysis. This approach, applied to datasets from healthy and obese individuals, improved peak detection and pattern realignment, enhancing the reliability of cross‐comparative analysis [138]. By using mass spectral total useful signal (MSTUS) and *Z*‐score normalization, the method enables more accurate identification of metabolic alterations, providing insights into disease‐related changes in saliva [196]. Similarly, a validated UHPLC–HRMS method provides comprehensive salivary metabolic profiling, demonstrating high linearity and precision for most metabolites and identifying 45 obesity‐related markers in adolescents. Complementarily, laser‐assisted rapid evaporative ionization MS enables rapid salivary fingerprinting with unique potential for point‐of‐care diagnostics. This integrated platform offers robust tools for exploring saliva's metabolome in health and disease [197].

Another essential strategy is expanding clinical cohorts to support the clinical translation of salivary metabolomics. Larger, more diverse patient populations will help ensure that findings are representative and applicable across different demographics, thereby improving the reliability of metabolite signatures. Currently, the majority of salivary metabolomics investigations remain in the discovery or early validation stages, with relatively modest cohort sizes. For instance, a study on CEG employed untargeted metabolomics in saliva from 64 patients and 30 controls, identifying 45 discriminative metabolites, of which seven exhibited AUC values greater than 0.80, suggesting excellent diagnostic potential despite limited sample size [29]. Similarly, an adolescent obesity study involving 140 participants reported salivary metabolic markers capable of differentiating obesity‐related phenotypes with high classification accuracy using multivariate models [197]. However, only a few studies have progressed to large‐scale, multicenter validations. Diagnostic performance across diseases remains variable, with reported AUC values typically ranging from 0.733 to 0.879 depending on disease type, population, and analytical model. For example, AUC values of 0.870 have been reported in CRC studies, while certain biomarkers for pSS achieved AUCs of 0.75–0.87 in validation cohorts [71, 125]. Although some machine‐learning models combining salivary metabolomics with other omics have reported sensitivities and specificities exceeding 90%, the lack of consistent external validation and standardized analytical pipelines continues to limit clinical implementation [86]. Therefore, expanding sample sizes, especially through multicenter collaborations, and standardizing protocols for cohort design and statistical analysis are critical next steps toward realizing the full diagnostic utility of salivary metabolomics.

Another key challenge in salivary metabolomics is the unclear temporal window it represents compared with other biofluids, such as blood and urine. While blood offers a real‐time snapshot of systemic metabolism, and urine reflects longer‐term metabolic fluctuations over hours or even days, the exact time frame that saliva captures remains uncertain [198, 199]. Saliva is likely influenced by both immediate and systemic factors, but the contribution of each is not well defined, making it difficult to interpret salivary metabolomic data in relation to disease states. Additionally, the mechanisms underlying the excretion of metabolites into saliva are not fully understood. While metabolites may enter saliva through passive diffusion, active transport, or overflow from systemic circulation, the relative importance of each pathway remains largely unexplored. This lack of clarity complicates the use of saliva for disease monitoring and biomarker discovery. To overcome these challenges, future research must focus on determining the time window captured by saliva and investigating the mechanisms of metabolite transfer. To achieve this, further research will be crucial in enhancing the utility of salivary metabolomics as a noninvasive diagnostic tool for systemic diseases, enabling more reliable and accurate insights into disease progression and biomarker identification [10, 200].

While saliva metabolic changes can signal the presence of a disease, the underlying pathophysiological processes driving these alterations are often not fully understood. The complexity of metabolic networks, coupled with the influence of various confounding factors, limits the capacity to link specific metabolic changes directly to disease mechanisms. To address these limitations and enhance the mechanistic insights provided by salivary metabolomics, integrating metabolomic data with complementary “omics” technologies—such as genomics, proteomics, and transcriptomics—will enable a more comprehensive understanding of the molecular mechanisms underlying disease states. Systems biology approaches that combine multiomics data can provide a holistic view of metabolic alterations within the context of gene expression, protein activity, and cellular signaling. This integrated approach allows for the identification of key regulatory nodes and metabolic pathways that are disrupted in disease, thereby providing more robust mechanistic insights [201]. Multiomic integration of proteomics, peptidomics, and targeted metabolomics in COVID‐19 salivary analysis reveals distinct biochemical alterations, identifying specific protein and peptide biomarkers. This approach uncovers the complex interplay between proinflammatory and anti‐inflammatory responses and highlights changes in amino acid profiles, offering high diagnostic efficiency and providing a comprehensive understanding of disease mechanisms [202]. Integration of electronic nicotine delivery systems (ENDS) emission profiles with salivary proteome and metabolome data through a multiomics approach revealed significant associations between metal exposure from e‐cigarette use and salivary biomarkers of inflammation and oxidative stress. This comprehensive analysis enhanced the accuracy of biomarker discovery and provided a more holistic understanding of the health risks associated with e‐cigarette use. Similarly, multiomics profiling of saliva from children with eating difficulties compared with healthy controls uncovered diminished antioxidant capacity and reduced levels of cystatins in the affected group.

To ensure the reliability and reproducibility of salivary metabolomics, it is essential to establish standardized protocols. While blood‐based metabolomics has well‐defined guidelines and is widely adopted in clinical practice, the lack of standardized procedures for saliva introduces significant variability that hinders comparisons across studies [203]. This variability arises from several factors, such as differences in collection methods, timing, and processing conditions, which can affect the integrity of salivary metabolite profiles. Consequently, the absence of universally accepted standards complicates the interpretation of results and limits the broader applicability of salivary metabolomics in clinical settings. In this context, referencing established frameworks, such as the NIH Metabolomics Workbench standards, could provide valuable guidance for harmonizing protocols across research groups. Emphasizing the importance of developing and adhering to standardized methodologies will ensure consistency in data collection and analysis, which is critical for the identification of robust biomarkers [204]. Furthermore, standardized practices would enable more accurate comparisons of results, strengthen the validity of salivary metabolomics as a diagnostic tool, and facilitate its integration into clinical workflows. The establishment of such standards will support the broader adoption of salivary metabolomics in both clinical diagnostics and systems biology research, enabling more reliable detection, monitoring, and understanding of systemic diseases (Table 4).

**TABLE 4:** Solutions to key challenges in salivary metabolomics clinical translation.

Challenge categories	Specific barriers	Proposed solutions	Specific examples	References
Standardization issues	Lack of standardized saliva collection protocols	Establishing consensus protocols for collection timing, method, and pretreatment	Adherence to frameworks like NIH Metabolomics Workbench	[204]
	Inconsistent processing and storage procedures	Defining and adopt standardized workflows for sample handling	Using of stabilizers; immediate freezing	N/A
Physiological uncertainty	Unclear temporal resolution of salivary metabolome	Clarifying the time frame saliva reflects through comparative studies	Paired saliva–blood–urine kinetics studies	[198, 199]
Analytical limitations	Difficulty detecting low‐abundance metabolites	Improving sensitivity and dynamic range of analytical platforms	UHPLC–HRMS; laser‐assisted REIMS	[197]
	Variability introduced by data acquisition and instrument fluctuations	Implementing robust normalization and strategies	MSTUS normalization; GC×GC–TOF MS with pattern realignment	[196]
	Complexity of salivary metabolite profiles hampers biomarker identification	Employing advanced data processing and multivariate analysis	Machine learning‐assisted biomarker screening	[184]
Mechanistic insight gaps	Limited interpretability of disease‐associated metabolic changes	Integrating metabolomics with other omics and applying network and pathway analysis for holistic understanding	Multiomics profiling in COVID‐19, ENDS exposure, pediatric feeding difficulties	N/A
	Poorly understanding metabolite excretion mechanisms	Investigating passive diffusion, active transport, and overflow pathways	In vitro models, in vivo tracing, molecular analysis, multiomics, and computational modeling	N/A
Clinical validation limitations	Small or demographically narrow clinical cohorts	Expanding sample sizes and population diversity	Progress toward multicenter validations	[71, 125]
	Limited external validation and reproducibility across studies	Adopting standardized study designs and performance metrics	Using of AUC, sensitivity/specificity, external validation cohorts	N/A
	Heterogeneity in diagnostic performance across diseases and analytical models	Optimizing biomarker panels and validation strategies for specific disease contexts	Machine‐learning based models combining salivary metabolomics with other omics	N/A

## 8 Conclusion and Prospects

Salivary metabolomics has established itself as a promising tool for the noninvasive detection and monitoring of systemic diseases. By providing a snapshot of both local and systemic physiological states, saliva offers advantages over traditional clinical samples, such as blood or urine, particularly in terms of accessibility, patient comfort, and repeatability. Technological advancements in MS, NMR spectroscopy, and other analytical platforms have significantly enhanced the sensitivity and specificity of salivary metabolomics, enabling the identification of biomarkers across a wide range of diseases, including metabolic disorders, cancers, and neurodegenerative conditions. However, despite these advancements, the translation of salivary metabolomics into routine clinical practice remains hindered by several technical and biological challenges.

One of the primary challenges facing salivary metabolomics is the inherent variability in saliva composition, influenced by factors such as sampling time, storage conditions, diet, lifestyle, and individual microbiome profiles. This variability complicates the identification of consistent and reliable biomarkers. Standardization of sample collection, handling, and analysis protocols is crucial to ensure data reproducibility and comparability across studies. While the potential of saliva as a diagnostic tool is clear, achieving consistent, high‐quality data across diverse clinical settings will require overcoming these preanalytical and technical challenges.

Looking ahead, integrating salivary metabolomics with multiomics approaches holds great potential for overcoming these limitations and enhancing the clinical utility of this diagnostic method. The combination of metabolomics with genomics, proteomics, and transcriptomics can provide a more comprehensive understanding of disease mechanisms, improving the specificity and sensitivity of diagnostic biomarkers. Furthermore, the application of advanced data processing techniques, including machine learning and artificial intelligence, can refine the analysis of large and complex datasets, enable the identification of novel biomarkers and improve the accuracy of disease predictions. Such integrative strategies may enable more precise monitoring of disease progression, therapeutic efficacy, and patient stratification for personalized treatments.

In addition to technological advancements, a deeper understanding of the physiological processes that govern metabolite transfer to saliva is essential for improving the interpretability of metabolomic data. The mechanisms through which blood‐borne metabolites enter saliva and how local factors—such as oral health and microbiome composition—affect the salivary metabolome remain areas of active research. By elucidating these pathways, we can better understand the disease‐specific alterations in the salivary metabolome, leading to the development of targeted biomarkers that are reflective of both systemic and oral health changes. This knowledge will strengthen the role of salivary metabolomics in precision medicine, providing an accessible, noninvasive tool for disease detection, monitoring, and personalized therapeutic strategies.

In conclusion, while salivary metabolomics has significant promise, its broader clinical adoption will depend on addressing current challenges related to standardization, data integration, and biological understanding. Future research focused on refining analytical techniques, exploring the biological mechanisms governing the salivary metabolome, and developing robust multiomics models will be essential for realizing the full potential of salivary metabolomics in clinical diagnostics. With these advancements, salivary metabolomics could become an invaluable tool for early detection and personalized management of systemic diseases, offering a cost‐effective and patient‐friendly approach to health monitoring.

## Author Contributions

L.C., X.Y., and S.H. conceived of the presented idea. L.C., X.Z., S.H., X.C., R.X., J.Z., Y.L., Y.Z., and Z.Z. wrote the manuscript. R.X., J.Z., Y.L., and Y.Z. created the graphs. All authors contributed to and approved the final manuscript.

## Ethics Statement

The authors have nothing to report.

## Consent

The authors have nothing to report.

## Conflicts of Interest

The authors declare no conflicts of interest.

## Data Availability

The data generated are included within the manuscript.
